# Modelling overflow metabolism in *Escherichia coli* with flux balance analysis incorporating differential proteomic efficiencies of energy pathways

**DOI:** 10.1186/s12918-018-0677-4

**Published:** 2019-01-10

**Authors:** Hong Zeng, Aidong Yang

**Affiliations:** Department of Engineering Science, University of Oxford, Parks Road, Oxford, OX1 3PJ UK

**Keywords:** Flux balance analysis, Proteomic efficiency, Overflow metabolism, Acetate production, Biomass yield, *Escherichia coli*

## Abstract

**Background:**

The formation of acetate by fast-growing *Escherichia coli* (*E. coli*) is a commonly observed phenomenon, often referred to as overflow metabolism. Among various studies that have been carried over decades, a recent work (Basan, M. et al. *Nature* **528,** 99–104, 2015) suggested and validated that it is the differential proteomic efficiencies in energy biogenesis between fermentation and respiration that lead to the production of acetate at rapid growth conditions, as the consequence of optimally allocating the limited proteomic resource. In the current work, we attempt to incorporate this newly developed proteome allocation theory into flux balance analysis (FBA) to capture quantitatively the extent of overflow metabolism in different *E. coli* strains.

**Results:**

A concise constraint was introduced into a FBA-based model with three proteomic cost parameters to represent constrained allocation of proteome over two energy (respiration and fermentation) pathways and biomass synthesis. Linear relationships were shown to exist between the three proteomic cost parameters. Tests with three different strains revealed that the proteomic cost of fermentation was consistently lower than that of respiration. A slow-growing strain appeared to have a higher proteomic cost for biomass synthesis than fast-growing strains. Different assumed levels of carbon flowing into pentose phosphate pathway affected the absolute value of model parameters, but had no qualitative impact on the comparative proteomic costs. For the prediction of biomass yield, significant errors that occurred for one of the tested strains (ML308) were rectified by adjusting the cellular energy demand according to literature data.

**Conclusions:**

With the aid of a concise proteome allocation constraint, our FBA-based model is able to quantitatively predict the onset and extent of the overflow metabolism in various *E. coli* strains. Such prediction is enabled by three linearly-correlated (as opposed to uniquely determinable) proteomic cost parameters. The linear relationships between these parameters, when determined using data from cell culturing experiments, render biologically meaningful comparative proteomic costs between fermentation and respiration pathways and between the biomass synthesis sectors of slow- and fast-growing species. Simultaneous prediction of acetate production and biomass yield in the overflow region requires the use of reliable cellular energy demand data.

**Electronic supplementary material:**

The online version of this article (10.1186/s12918-018-0677-4) contains supplementary material, which is available to authorized users.

## Background

The formation of acidic by-products, predominantly acetate, when *Escherichia coli* (*E. coli*) grows under aerobic-glucose conditions is a commonly observed phenomenon, which has been extensively studied over decades [1–5]. Lee reviewed 19 studies of recombinant *E. coli* where acetate was accumulated in fed-batch systems [6]. It has been reported that the portion of glucose converted into acetate can be as high as 15% [7], representing a seemingly huge waste of feedstock. The accumulation of acetate in the culture medium appears to be a major limiting factor for achieving high cell density [8], which is particularly severe in the growth of recombinant strains [9]. Acetate also impairs the microbial production of recombinant proteins [1] and drug precursors [9]. These complications of acetate in bioreactors thus call for elucidation of acetate-pertinent metabolic processes. A similar phenomenon has been observed in tumour cells (Warburg effect) [10–12]. The associated mathematical models for explaining the Warburg effect have recently been reviewed [13].

Traditionally, the aerobic formation of acetate has been referred to as overflow metabolism: the excess glucose saturates or inhibits the tricarboxylic acid (TCA) cycle, which subsequently forces the cell to modulate the redundant carbon to the acetate pathway [3, 14]. However, the study by Molenaar et al. suggested that the overflow metabolism as shown in the growth phenotype is probably a result of the global allocation of cellular resources, where the enzyme efficiency and the pathway yield were both taken into account to obtain the optimal growth strategies subject to different growth conditions [15]. Later in 2015, Basan et al. proposed and validated that the overflow metabolism in *E. coli* originates from the global physiological proteome allocation for rapid growth [16]. In particular, the proteomic efficiency of energy biogenesis through aerobic fermentation was found to be higher than that of respiration; this difference in proteomic efficiency between fermentation and respiration appears to play a central role in dictating the degree of overflow metabolism in *E. coli*.

Given the importance of the overflow metabolism, several phenomenological models were developed to depict this effect [5, 8, 17, 18], where the prediction of acetate excretion was dictated by a combination of (i) the constraints on oxygen and carbon supply and (ii) cellular mass and energy balance. Later, models that adopt conventional regulatory mechanisms of acetate metabolism [14], such as oxygen limitation, carbon source availability and tight regulation of cofactor pools were evaluated [19], with an attempt to explain the metabolic shifts from a fully aerobic mode to the aerobic acetate fermentation (overflow). More recently, constraint-based metabolic models [20] were established to analyse the optimal cellular growth strategy, incorporating principles of (i) limitation in the cellular resource on the maximal attainable growth rate, such as the maximum cytoplasmic density adopted by FBAwMC [21, 22] and the finite amount of resource to be allocated between metabolic network and ribosomes, as applied in RBA [23–25], (ii) metabolic regulation based on enzyme kinetic information, such as mechanistically detailed descriptions of gene expression and the synthesis of functional macromolecules used in ME-Model [26] and (iii) membrane occupancy-derived competition between glucose transporters and respiration chain (an extension of ME-Model) [27]. The major target of these models is to predict the maximum cellular growth rate. Predictions were validated quantitatively by the experimental data, while the overflow metabolism in fast-growing phase was mostly captured in a qualitative way. In addition, it was pointed out [16, 28] that cell volumes were empirically found to vary widely with virtually constant densities across different growth conditions [29], which suggests that the cytoplasmic density-based constraint might not be fully justified.

Inspired by a recent experimental work studying the proteomic cost of the core metabolic pathways of *E. coli* [16], a model named constrained allocation flux balance analysis (CAFBA) [28] managed to predict the rates of acetate production in the overflow metabolism for different *E. coli* strains, with good quantitative agreement with experimental data. However, the proteomic costs adopted in CAFBA were applied to individual metabolic reactions, without focusing on the exploration of the critical role played by specific metabolic modules such as energy biogenesis pathways.

In this work, we attempt to depict the overflow metabolism in various *E. coli* strains with quantitative accuracy, i.e. predicting aerobic steady-state rates of acetate production at different growth rates and validating the model with experimental data in literature. In particular, we adopt a concise proteome allocation constraint as identified by Basan et al. [16], referred to as the Proteome Allocation Theory (PAT) in this work. The PAT suggests that the choice of energy biogenesis pathways under different growth conditions results from the discrepancy of proteomic efficiencies between fermentation and respiration. *E.coli* cells tend to use the more protein-efficient fermentation pathway to generate energy in order to accommodate the high proteomic demand in biosynthesis under rapid growth. The key concepts of PAT are fully embedded and realised in our model. With a parsimonious, PAT-based metabolic model capable of accurately capturing the overflow metabolism, we further analyse the interdependency between pathway-level proteomic cost parameters, the disparity in these parameters between different *E. coli* strains, and the impact of cellular energy demand on the accuracy of the co-prediction of the overflow metabolism and the biomass yield on substrate.

## Methods

### Formulation of the PAT constraint

Following Basan et al. [16], the fractions of three proteome sectors in the entire proteome (i.e. the total protein content) of the cell sum to unity:

1 $$ {\phi}_f+{\phi}_r+{\phi}_{BM}=1 $$

where *ϕ*_*f*_ and *ϕ*_*r*_ are the fractions of the fermentation- and respiration-affiliated enzymes, respectively, which enable the fluxes for energy generation; *ϕ*_*BM*_ represents the fraction of the remaining part of the proteome enabling other cellular activities, broadly referred to as the sector of biomass synthesis [16, 28].

More specifically, *ϕ*_*f*_ represents the mass abundance of the enzymes that carry fermentation fluxes involved in glycolysis (glucose to acetyl-CoA), oxidative phosphorylation and acetate synthesis pathways (phosphotransacetylase and acetate kinase). *ϕ*_*r*_ comprises all the enzymes that catalyse the respiration-associated reactions in glycolysis, tricarboxylic acid (TCA) cycle and oxidative phosphorylation system. Same as in Basan et al. [16], in this work we adopt the linear dependences assumed in Hui et al. [30] to relate *ϕ*_*f*_ and *ϕ*_*r*_ with the fermentation and respiration fluxes respectively,

2a $$ {\phi}_f={w}_f{v}_f $$

2b $$ {\phi}_r={w}_r{v}_r $$

where *v*_*f*_ (*v*_*r*_) is the fermentation (respiration) pathway flux, which in this work is represented by the enzymatic reaction “acetate kinase ACKr” (“2-oxogluterate dehydrogenase AKGDH”); *w*_*f*_ (*w*_*r*_) is the pathway-level proteomic cost, denoting the proteome fraction required per unit fermentation (respiration) flux.

On the biomass synthesis sector, *ϕ*_*BM*_ corresponds to the remaining part of proteome that is not covered by the fermentation and respiration sectors, including ribosomal proteins and anabolic enzymes (the major part, referred to as biomass synthesis), catabolic enzymes and cellular maintenance proteins. Motivated by the observed linear dependency between growth rate and proteome fraction for biomass synthesis [30–32], the following linear relationship is assumed:

3 $$ {\phi}_{BM}={\phi}_0+b\uplambda $$

where *b*λ is the growth rate-associated component with λ being the specific growth rate and the constant *b* quantifying the proteome fraction required per unit growth rate. In Basan et al. [16], *ϕ*_0_ was considered as a growth rate independent constant.

Combining Eqs. (1)–(3), we have

4 $$ {w}_f{v}_f+{w}_r{v}_r+b\uplambda =1-{\phi}_0 $$

Equation (4) implies that the sum of the three proteomic cost terms on the left-hand side remains constant. However, when the growth rate (and hence the fermentation and respiration fluxes) becomes very low, it is difficult to envisage numerically how this sum could still remain at a constant level. In fact, in Basan et al. [16] (see its Supplementary Information), it was acknowledged that at growth rates lower than that corresponding to the onset of the overflow phenomenon, the proteome sectors would no longer be constrained by the equality indicated by Eq. (4). This suggests that across the entire range of possible growth rates, *ϕ*_0_ is unlikely a growth rate independent constant: it may remain at a constant (and minimum) level in the overflow region where the proteomic resource is stretched, but become growth-rate dependent (and larger) at lower growth rates outside the overflow region, i.e. *ϕ*_0, min_ ≤ *ϕ*_0_ ≤ 1, where *ϕ*_0, min_ is a true constant. Defining *ϕ*_max_ ≡ 1 − *ϕ*_0, min_, Eq. (4) then becomes

5 $$ {w}_f{v}_f+{w}_r{v}_r+b\uplambda =1-{\phi}_0\le 1-{\phi}_{0,\min}\equiv {\phi}_{\mathrm{max}} $$

In Vazquez and Oltvai (2016) [33], *ϕ*_0_ was also interpreted as a variable instead of a constant, with a (non-zero) minimum value. For simplicity, both sides of Eq. (5) is divided by *ϕ*_*max*_, leading to the final form of the proteome constraint adopted in this work, referred to as PAT constraint from this point on:

6 $$ {w}_f^{\ast }{v}_f+{w}_r^{\ast }{v}_r+{b}^{\ast}\lambda \le 1 $$

where ${w}_f^{\ast}\equiv {w}_f/{\phi}_{max}$, ${w}_r^{\ast}\equiv {w}_r/{\phi}_{max}$ and *b*^∗^ ≡ *b*/*ϕ*_*max*_. ${w}_f^{\ast }$, ${w}_r^{\ast }$ and *b*^∗^ are referred to as the proteomic cost parameters.

### Predicting the acetate flux

Flux balance analysis (FBA) [20] is used to determine the optimal flux distribution under different growth condition, with a set of constraints:

max*f*_*obj*_, subject to

7 $$ {\displaystyle \begin{array}{l}\left(\mathrm{i}\right)\kern0.5em \mathrm{Sv}=0\\ {}\left(\mathrm{i}\mathrm{i}\right)\kern0.5em {\mathrm{v}}^{\mathrm{L}}\le \mathrm{v}\le {\mathrm{v}}^{\mathrm{U}}\\ {}\left(\mathrm{i}\mathrm{i}\mathrm{i}\right)\kern0.5em {w}_f^{\ast }{v}_f+{w}_r^{\ast }{v}_r+{b}^{\ast}\lambda \le 1\end{array}} $$

where *f*_*obj*_ is the assumed cellular objective. We specified minimizing substrate uptake as the objective function because in this study the commonly used objective ‘growth rate’ was used as the model input (with acetate production as the model output). S is the stoichiometric matrix defined by the metabolic model; v is a column vector comprising the reactions/fluxes described in the metabolic network; v^L^ and v^U^ represent the lower and upper limits of the reactions, respectively. The inequality constraint (iii) is same as Eq. (6) introduced earlier.

The prediction of the extent of overflow metabolism (rate of acetate production) requires of the parameter values of ${w}_f^{\ast }$, ${w}_r^{\ast }$ and *b*^∗^ in the third constraint (PAT constraint) of Eq. (7). We show in the next section that these three parameters cannot be uniquely determined by the experimentally measured growth rate-acetate production profile alone. A set of values for these parameters was randomly chosen from mathematically equivalent sets (see Additional file 1: Table S1). The PAT-based FBA was run at different growth rates under aerobic-glucose conditions. FBA was carried out using the core *E. coli* metabolic model [34], referred to as the core model in the rest of the paper. The optimal flux distribution was solved via COBRA toolbox [35] in MATLAB (R2016a). LP solution was determined by Gurobi 6.0. Detailed model descriptions such as uptake bounds and flux regulations are given in Additional file 2, sections 1 and 2.

### Interdependency of proteomic cost parameters

A linear relationship was previously shown to hold between the fermentation (or respiration) flux and steady state growth rates in the overflow region [16]:

8 $$ {v}_f={k}_f\lambda +{v}_{f,0} $$

9 $$ {v}_r={k}_r\lambda +{v}_{r,0} $$

where, as introduced earlier, *v*_*f*_ is the fermentation flux (referred to as “acetate line” in [16]); *v*_*r*_ is the respiration flux. *k*_*f*_ (*k*_*r*_) and *v*_*f*, 0_ (*v*_*r*, 0_) are constants representing the slope and intercept of the fermentation (respiration) line. Substituting Eq. (8) and Eq. (9) into Eq. (6), with the equal sign held for the overflow condition:

10 $$ \left({w}_f^{\ast }{k}_f+{w}_r^{\ast }{k}_r+{b}^{\ast}\right)\lambda =1-{w}_f^{\ast }{v}_{f,0}-{w}_r^{\ast }{v}_{r,0} $$

Equation (10) holds for any growth rate (*λ*) in the overflow region, which requires

11 $$ {w}_f^{\ast }{k}_f+{w}_r^{\ast }{k}_r+{b}^{\ast }=0 $$

12 $$ 1-{w}_f^{\ast }{v}_{f,0}-{w}_r^{\ast }{v}_{r,0}=0 $$

Equations (11) and (12) indicate that (i) there is a linear relationship between the fermentation and respiration proteomic cost parameters ${w}_r^{\ast }$ and ${w}_f^{\ast }$, and (ii) the third growth-rate dependent proteomic cost parameter *b*^∗^ is a linear combination of ${w}_r^{\ast }$ and ${w}_f^{\ast }$, thus *b*^∗^ also possesses a linear relationship with ${w}_f^{\ast }$ (or ${w}_r^{\ast }$).

If experimental data exist that allow for both the fermentation line and the respiration line to be plotted (such as the steady state growth rate – acetate excretion and growth rate – CO_2_ revolution data given in [16]), their slopes and intercepts, appearing in Eqs. (11) and (12), can be obtained. However, the three proteomic cost parameters cannot be uniquely determined by the two equations, although specific values of similar parameters have previously been derived from measured cellular protein compositions [16].

**Fig. 1:**
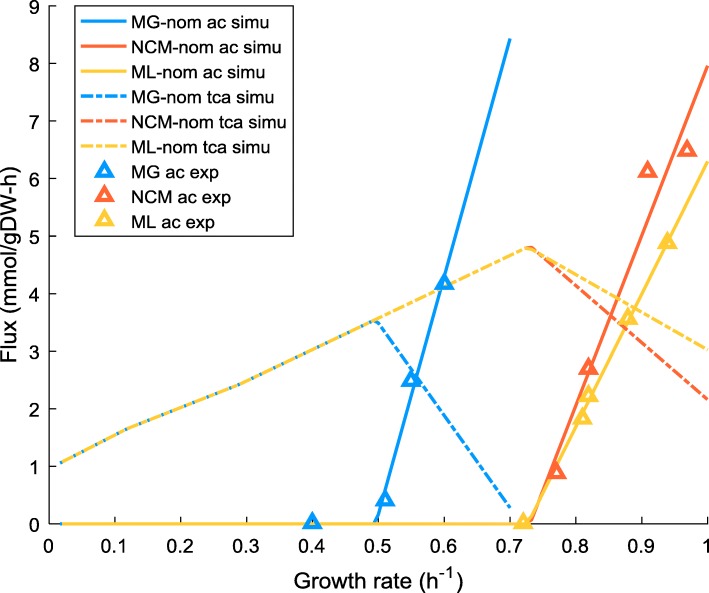
Model predictions of overflow metabolism for MG, NCM and ML at nominal energy demand. The extent of overflow metabolism is represented by the acetate flux. Simulation results of the respiration flux are drawn to show the switch between fully-respiration and respiration-fermentation mode. Comparison is made between model predictions and experimental data for the rates of acetate production. *uPPP*% was set to 35% according to the flux measurement [41]. Other *uPPP*% values render similar results (see Additional file 1: Table S1 and Additional file 2, section 3). Experimental data were obtained from different sources [3, 16, 41]. Data points from [16] were converted using 1 mM A_600nm_^− 1^ h^− 1^ = 2 mmol gDW^− 1^ h^− 1^ according to [28]. “-nom” refers to nominal, the default energy demand specified in the core model. ac – acetate flux, vr – respiration flux, simu – simulation results, exp. – experimental data

In this work, *k*_*f*_ and *v*_*f*, 0_ were directly determined from the experimentally measured growth rate-acetate excretion profile (data sources are shown in Fig. 1). To our knowledge, no directly experimental data were available for the rate of intracellular respiration. Alternatively we took the growth rate-acetate profile as the input of FBA (setting the objective function to the minimisation of glucose uptake) to estimate the respiration flux at each data point, which was subsequently used to determine *k*_*r*_ and *v*_*r*, 0_. Flux variability analysis (FVA) was conducted which confirmed that all the relevant fluxes used in the model were uniquely determined. After obtaining *k*_*f*_ (*k*_*r*_) and *v*_*f*, 0_ (*v*_*r*, 0_), a set of values of ${w}_f^{\ast }$, ${w}_r^{\ast }$ and *b*^∗^ can be determined by arbitrarily specifying the value for one of the parameters. In this work, we took ${w}_f^{\ast }$ to be specified, in a range of [0, 0.11] for MG1655 and [0, 0.07] for ML308 and NCM3722. This thus allows us to present the parameter estimation results in the form of ${w}_f^{\ast }$-${w}_r^{\ast }$ and ${w}_f^{\ast }$-*b*^∗^ plots. Simulation results presented in this paper were obtained with a randomly chosen value of ${w}_f^{\ast }$ within the ranges mentioned above and the correspondingly determined values of ${w}_r^{\ast }$ and *b*^∗^. Note that different values chosen for ${w}_f^{\ast }$ yielded identical simulation results (Additional file 2: Figures S9-S17).

### Adjustment of cellular energy demand

We observed discrepancy in biomass yield between model predictions and the experimental data, especially for ML308 (Fig. 5), and hypothesised the reason being the inaccuracy of cellular energy demand assumed by the core model when applied to this particular species growing under non-overflow and overflow conditions. To test this hypothesis, we collected the growth data in the overflow region for ML308 (Table 7 in [3]) and found that the reported steady state ATP production rate was lower than what the core model suggested. Therefore, we decided to remodel the cellular energy demand by subtracting the surplus portion, reducing it to the strain-specific values reported in the literature (Additional file 1: Table S3).

The original cellular energy demand embedded in the core model is quantified by

13 $$ {r}_{ATP, nominal}=\mathrm{ATPM}+\sigma \lambda +{v}_{GLNS}+{v}_{PFK} $$

where *r*_*ATP*, *nominal*_ is the overall ATP consumption rate (equivalent to the ATP production rate at steady state); subscript “nominal” indicates the default specification of the core model; *v*_*GLNS*_ and *v*_*PFK*_ are the fluxes of the enzymatic reactions glutamine synthetase and phosphofructokinase (one mole ATP is required per mole flux of each reaction); (ATPM + *σλ*) denotes the maintenance energy required for non-metabolic processes, where ATPM corresponds to the non-growth-associated maintenance (NGAM) and *σ* to the growth-associated maintenance (GAM) [36].

The adjusted cellular energy demand is formulated as

14 $$ {r}_{ATP, new}={r}_{ATP, nominal}-S\left(\lambda \right) $$

where *S*(*λ*) is the offset energy, i.e. the amount of energy over-predicted by the core model. The mathematical analysis of the growth data of ML308 suggested that in the overflow region, the offset energy is linearly related with the growth rate (R^2^ = 0.9998):

15 $$ S\left(\lambda \right)= k\lambda +c $$

where *k* and *c* are constants. Substituting Eqs. (13) and (15) into Eq. (14), we have

16 $$ {r}_{ATP, new}=\left(\mathrm{ATPM}-c\right)+\left(\sigma -k\right)\lambda +{v}_{GLNS}+{v}_{PFK} $$

For simplicity, we define M ≡ ATPM − *c* and N ≡ *σ* − *k*; M and N are referred to as the (adjusted) maintenance parameters. The adjustment of the cellular energy demand in our FBA is achieved by manipulating the maintenance energy, more specifically, through the maintenance parameters. Note that in the case where growth energetic data is not available (i.e. for MG1655 and NCM3722), such adjustment was not possible, therefore the default maintenance parameter values were used (see Table 1).

**Table 1:** Values of the maintenance parameters

Strains	M	N	Scope of applicability
MG-/NCM−/ML-nom	8.39^a^	59.81^a^	Complete growth range
ML-new	− 50.90^b^	93.34^b^	Overflow region

^a^default value specified in the *E. coli* core model

^b^estimated from the growth data (Table 7 in Holms 1996 [3])

### Alternative pathways in central metabolism

The model constructed in this work considers only the central metabolism of *E. coli* as detailed in the *E. coli* core model. The energy biogenesis pathways in the model consist of glycolysis (the EMP pathway), the TCA cycle, the acetate pathway (PTA-ACKA) and the terminal oxidative phosphorylation system. However we noted the existence of alternative pathways in the central carbon metabolism, which include the Entner-Doudoraff (ED) pathway, the pentose phosphate (PP) pathway and the more recently explored PEP-glyoxylate cycle [37].

### ED pathway

The ED pathway was found to be three to five-fold more protein-efficient than the EMP pathway to achieve the same glycolytic flux [38], which provides a clear rationale for the utilisation of the ED pathway in a number of bacteria, e.g. *Sinorhizobium meliloti, Rhodobacter sphaeroides, Zymomonas mobilis, and Paracoccus versutus* [39]. However, Flamholz et al. acknowledged that *E. coli*, which is capable of using both the ED and the EMP pathways, tends to use the latter. Flux measurements also suggest that the usage of the ED pathway by *E.coli* K-12 is minimal: only about 2% of glucose catabolism proceeds by means of the ED pathway in batch cultures [40] and about 6% in mini-scale chemostats [41]. Furthermore, the activity of the ED pathway was detected only under slow- to mild-growing conditions [40, 41]. To our knowledge, no activity of ED pathway in *E. coli* has been reported under fast-growing scenarios.

### PEP-glyoxylate cycle

As for the PEP-glyoxylate cycle, similar to the ED pathway, its usage was identified to be significant only under slow-growing conditions. Not even a trace activity of the PEP-glyoxylate cycle was found in wild-type batch cultures or more rapidly growing chemostats [37]. Furthermore, the flux comparison between the aceA-pckA knockout strain and the sucC knockout strain [16] verifies that compared to the TCA cycle, the alternative PEP-glyoxylate cycle plays a less significant role in glucose-limited fast-growing cultures of *E. coli*.

Based on above literature evidences, this work, focusing on the overflow metabolism that occurs at fast-growing cultures of *E.coli* with relatively sufficient substrate availability, has taken the assumption that the use of alternative ED pathway and PEP-glyoxylate cycle is negligible compared to the glycolysis (i.e. EMP pathway) and the TCA cycle.

### PP pathway

The PP pathway, on the other hand, can function as a significant alternative to the upper part of glycolysis for carbon catabolism in *E. coli*. Previous studies showed that the carbon flow through the PP pathway could reach 20–35% of the total carbon intake and can vary with different growth rates [40, 41] hence neither a constant portion of carbon is diverted into PP pathway nor this portion of carbon flux negligible. The uncertainty embedded in the PP pathway flux motivates us to study the impact of different portion of substrate carbon allocated between the upper part of the EMP pathway and the PP pathway on the proteomic cost parameters and the model predictions. More details can be found in the Additional file 2, section 3.

We define the PP pathway ratio (*PPP*%) as the portion of substrate carbon directing to PP pathway to the total carbon intake:

17 $$ PPP\%=\frac{PGL}{EX\_ glc\left(\mathrm{e}\right)}\times 100\% $$

where 6-phosphogluconolactonase (PGL) is chosen to represent the PP pathway flux as it is a major and also the beginning enzymatic reaction in the pentose phosphate shunt; EX_glc(e) is the exchange reaction denoting glucose uptake rate. In our simulation, *PPP*% was controlled by setting the upper bound of the portion of carbon that is directed into the PP pathway, denoted as *uPPP*%, with the aid of an auxiliary term DM_PPP_RATIO:

18 $$ uPPP\%\times EX\_ glc(e)- PGL= DM\_ PPP\_ RATIO\ge 0 $$

## Results

*E.coli* MG1655, NCM3722 and ML308 have been selected as the model strains in this work and are referred to as MG, NCM and ML respectively from this point on. In this section, the simulated acetate excretion pattern is presented against experimental data to demonstrate the accuracy of the model prediction. Subsequently we elucidate the linear interdependency of the proteomic cost parameters. In particular, we reveal the similarities and differences between the three *E. coli* strains. With respect to the PP pathway ratio (*PPP*%), previous studies on the slow-growth strain MG show that the portion of carbon that goes into the PP pathway can be approximately 20% of the total carbon intake [40, 41]. In this work, we set the upper bound of the carbon flowing into PP pathway (*uPPP*%) to 25, 35 and 40% to investigate the potential effect of the change in *PPP*% on proteomic cost parameters and model prediction (more justification is provided in Additional file 2, section 3). On cellular energy demand, we refer to the original energy demand specified in the core model [34] as nominal energy demand, and present first the set of results which were generated on this basis. Subsequently, we show how an adjusted energy demand (particularly applied to ML, referred to as ML-new) affects the patterns of the estimated proteomic cost parameters and the accuracy of biomass yield prediction.

### Model prediction of overflow metabolism with nominal energy demand

Here, the accuracy of the predicted acetate excretion rate is compared with experimental data. Variation in the proteomic cost parameters with the changed carbon level diverted into the PP pathway is also presented.

### Model prediction of acetate production

Figure 1 shows that model prediction of the pattern of acetate excretion is in good agreement with the experimental observations for three different *E. coli* strains. The onset of the production of acetate is concomitant with the drop in the respiratory flux, indicating a switch between fully-respiration to respiration-fermentation mode. As the growth rate further increases, the acetate flux becomes dominant while the extent of respiration is gradually diminishing. It is worth noting that zero acetate production was commonly observed at low growth rates of different strains [3, 16, 41, 42]; To emphasise the (strain-specific) acetate production pattern, we only collect the data with non-zero acetate production. For all the strains, data involving growth rates lower than those presented in Fig. 1 are associated with non-detectable acetate excretion, hence are not shown here.

### Linear relationships between proteomic cost parameters

**Fig. 2:**
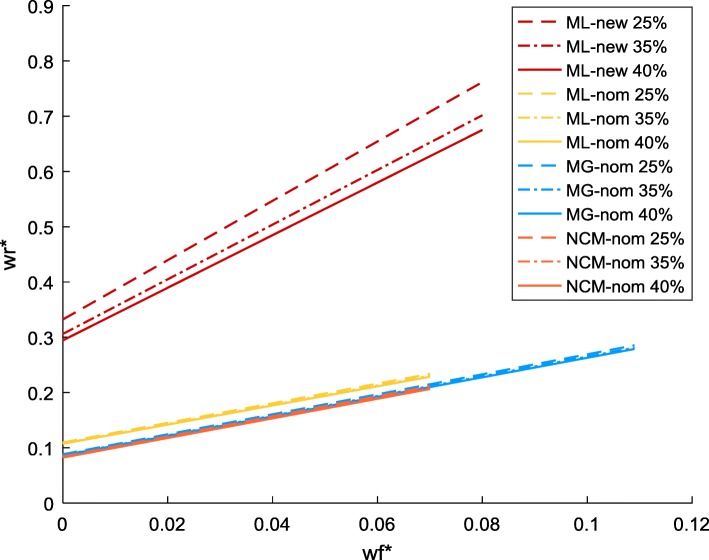
${w}_r^{\ast }-{w}_f^{\ast }$ relationship for MG, NCM and ML with nominal energy demand and for ML with new energy demand. “-nom” refers to nominal, the default energy demand specified in the core model. “-new” refers to the adjusted energy demand. *uPPP*% was set to 25, 35 and 40% for each strain

When the nominal energy demand is adopted (indicated by “-nom”), the change of *uPPP*% leads to insignificant changes to the ${w}_r^{\ast }-{w}_f^{\ast }$ line for each strain (Fig. 2). Between different strains, MG and NCM share nearly identical lines. The lines of ML-nom deviate from those of the former two, but not significantly (although this closeness will be altered with the adjusted energy demand, see Fig. 2 ML-new and the section below). In any case, ${w}_r^{\ast }$ is clearly higher than the corresponding ${w}_f^{\ast }$, implying that respiration has a higher (lower) proteomic cost (efficiency) than fermentation for energy production, which is consistent with what was derived from protein abundances data for comparable parameters in [16].

To inspect the insignificant disparity in the ${w}_r^{\ast }-{w}_f^{\ast }$ lines when all strains use the nominal energy demand, Eq. (12) is re-arranged to

19 $$ {w}_r^{\ast }=\frac{v_{f,0}}{v_{r,0}}{w}_f^{\ast }+\frac{1}{v_{r,0}} $$

The slope and intercept of the ${w}_r^{\ast }-{w}_f^{\ast }$ line are dictated by $\frac{v_{f,0}}{v_{r,0}}$ and $\frac{1}{v_{r,0}}$, respectively. *v*_*f*, 0_ can be determined directly by the experimental measurement of acetate production. *v*_*r*, 0_, on the other hand, is a result of the combination of (measured) rates of acetate production and the mass and energy balance structure of the metabolic model.

For a specific strain, *v*_*f*, 0_ only depends on the pattern of acetate excretion, not affected by assumed level of *uPPP*%. Therefore, the impact of *uPPP*% on the ${w}_r^{\ast }-{w}_f^{\ast }$ line is through affecting the value of *v*_*r*, 0_, which turns out to be rather moderate. Between different strains, the ratio of *v*_*f*, 0_ and *v*_*r*, 0_ and the value of *v*_*r*, 0_ are nearly identical between MG and NCM, regardless of the level of *uPPP*% adopted, resulting in the very much overlapped pattern of the ${w}_r^{\ast }-{w}_f^{\ast }$ relationship between MG and NCM. For ML, the value of the slope is slightly smaller than MG and NCM, while the intercept is about 25% larger (as shown in Additional file 1: Table S2). Figure 2 also suggests that the proteomic cost (efficiency) of respiration pathways for ML is higher (lower) than that for MG and NCM, regardless the modification in the energy demand.

**Fig. 3:**
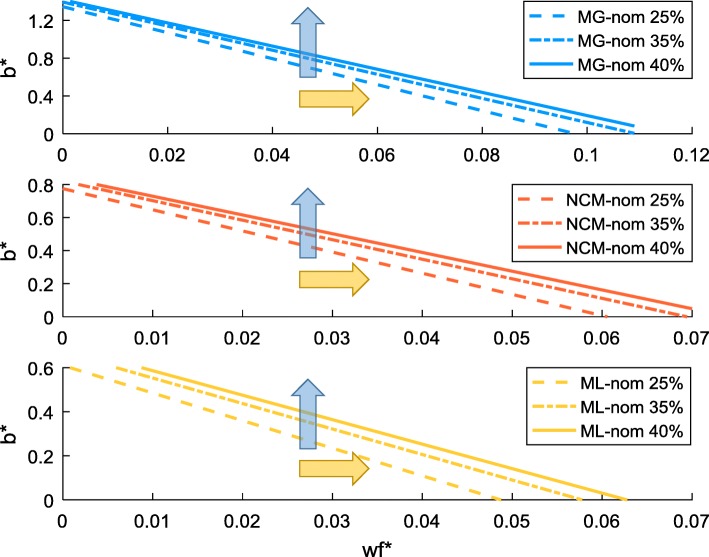
${b}^{\ast }-{w}_f^{\ast }$ relationship for MG, NCM and ML strains with nominal energy demand. “-nom” refers to nominal, the default energy demand specified in the core model. *uPPP*% was set to 25 35 and 40% for each strain. The blue arrow shows the increase in *b*^∗^ with the increase of *uPPP%* at fixed ${w}_f^{\ast }$; the yellow arrow shows the right-shifting trend of the ${b}^{\ast }-{w}_f^{\ast }$ line with the increase of *uPPP%*

Compared to the ${w}_r^{\ast }-{w}_f^{\ast }$ relationship, that of ${b}^{\ast }-{w}_f^{\ast }$ appears to be affected by the level of *uPPP*% more visibly (Fig. 3). Between different species, the difference is also more pronounced, and closeness is present between the two rapid-growth strains NCM and ML (as presented in Additional file 2: Figure S1).

For a specific strain, the increase of *uPPP*% gradually moves the ${b}^{\ast }-{w}_f^{\ast }$ line to the right (yellow arrow, Fig. 3), corresponding to an increase in *b*^∗^ (blue arrow, Fig. 3). This trend can be explained by inspecting a re-arrangement of Eq. (11):

20 $$ {b}^{\ast }=\left({k}_r\frac{v_{f,0}}{v_{r,0}}-{k}_f\right){w}_f^{\ast }+\frac{k_r}{v_{r,0}} $$

Equation (20) suggests that the shift of the ${b}^{\ast }-{w}_f^{\ast }$ line results from the change in the respiratory flux (note the intercept, $\frac{k_r}{v_{r,0}}$). In *E. coli*, the PP pathway and the TCA cycle are two major sources for the production of NADPH [40]. At a given growth rate, the amount of NADPH needed for cell growth is fixed based on the mass and energy balance. As *uPPP*% increases, more carbon is predicted to enter the PP pathway. In the model simulation, an increase in the amount of NADPH produced via PP pathway would force a drop of the flux into the TCA cycle in order to maintain the constant total production rate of NADPH. The reduction in the TCA flux in turn manifests in a lower *v*_*r*_. In the overflow region, Eq. (6) is bounded by the equal sign. As shown earlier, the level of *uPPP*% has a negligible impact (when nominal energy demand is adopted) on the ${w}_r^{\ast }-{w}_f^{\ast }$ line. Also recall that the relation between the rate of acetate excretion and steady state growth rate is fixed by the experimentally measured growth data. With all the other quantities (${w}_f^{\ast }$, *v*_*f*_, ${w}_r^{\ast }$ and *λ*) fixed in Eq. (6), the drop in *v*_*r*_ due to the increase in *uPPP*% will necessarily be accompanied by an increase in *b*^∗^.

Between different strains, *b*^∗^ varies significantly. In particular, *b*^∗^ for MG is remarkably larger than that of NCM and ML (as presented in Additional file 2: Figure S1). This disparity can again be explained by Eq. (6). For a certain value of ${w}_f^{\ast }$, ${w}_r^{\ast }$ is rather similar among different strains (with nominal energy demand) as shown by Fig. 2. In the overflow region, the respiration flux *v*_*r*_ of MG is much smaller than the others (see Fig. 1), which thus leads to a lower value of the ${w}_r^{\ast }{v}_r$ term for MG than NCM and ML. As the value of the ${w}_f^{\ast }{v}_f$ term (for any selected value of ${w}_r^{\ast }$) is similar between these strains, due to their similarity in the relationship between ${w}_r^{\ast }$ and ${w}_f^{\ast }$, the value of the remaining term on the left-hand side of Eq. (6), *b*^∗^*λ*, must be higher for MG than for the other two strains. On the other hand, in the overflow region and at a same acetate excretion rate *v*_*f*_, the growth rate of MG has been shown to be much lower than that of NCM and ML. Now, a higher value of *b*^∗^*λ* coupled with a lower value of *λ* will undoubtedly lead to a higher value of *b*^∗^for MG, compared to the other two strains.

The above mathematical explanation in fact coincides with the known biological fact that the inverse of *b*^∗^ is proportional to the rate of protein synthesis [31]: the slower the rate of protein synthesis, the higher the value of *b*^∗^. Thus for the slow-growing strain MG, it is expected to have a higher value of *b*^∗^compared to the fast-growing strains NCM and ML.

### Predicted evolution of PP pathway flux

The results presented above show rather moderate impact of the upper limit of PP pathway ratio (*uPPP*%) on the linear interdependency of the proteomic cost parameters. With an interest in the FBA solution of the flux distribution in PP pathway (at different growth rates), simulation results were recorded for three strains with *uPPP*%set to 35%; other *uPPP%* levels displayed a similar trend (as presented in Additional file 2: Figures S4 and S5). Flux variability analysis (FVA) [43] was performed to confirm that the trend of *PPP*% presented here was unique.

**Fig. 4:**
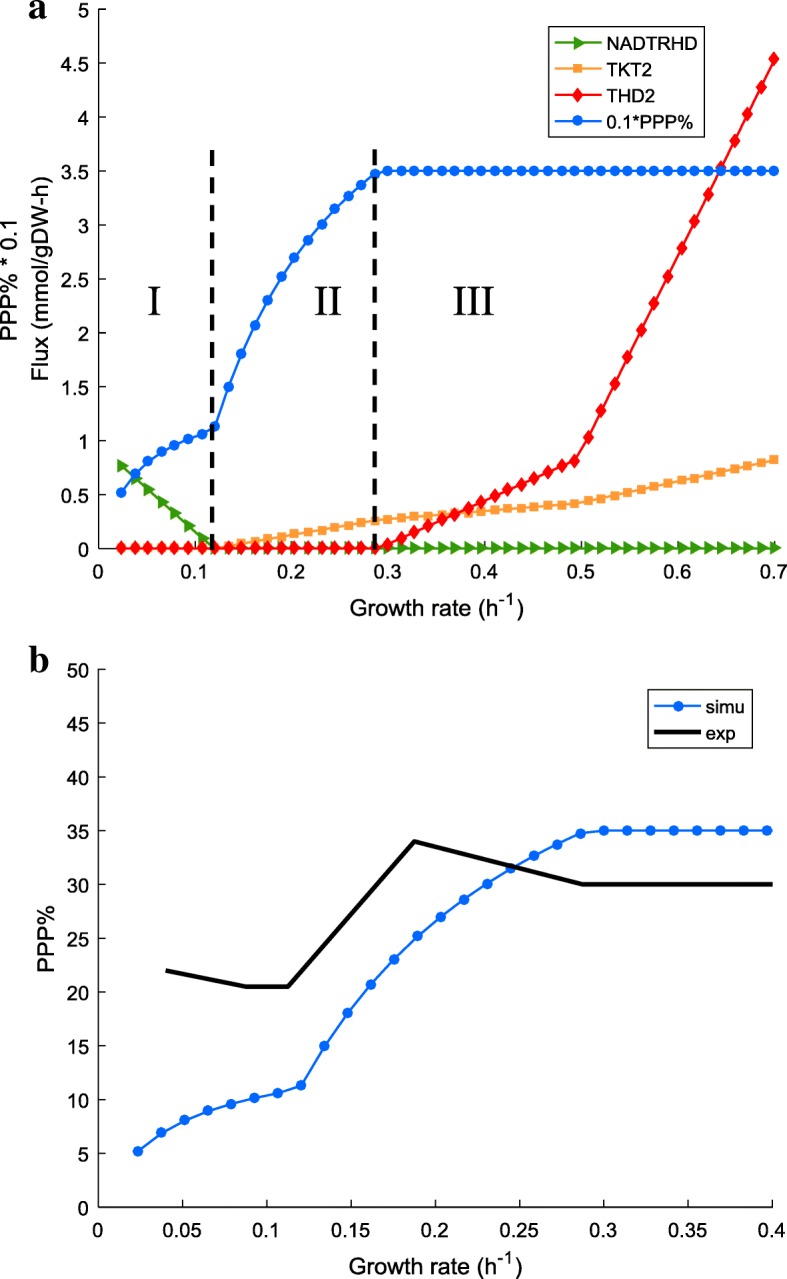
**a** Simulation results of *PPP*%, NADTRHD, TKT2 and THD2 against growth rates at nominal energy demand. **b** Comparison between predicted trend of *PPP*% and experimental data. PP pathway ratio (*PPP*%) is divided by ten (0.1*PPP%) to unify the order of magnitude between different data types. Experimental data were obtained from [41]. *uPPP*% was set to 35%. Simulation was based on MG1655. NADTRH – NAD transhydrogenase, TKT – transketolase, THD2 – NADP transhydrogenase, simu – simulation results, exp. – experimental data

In general, *PPP*% gradually increases with the growth rate. Two turning points can be observed, which divide the whole curve into three distinct phases (Fig. 4a). A close inspection of the model simulation revealed that the variation of the predicted PP pathway ratio was co-related particularly with three fluxes, namely NAD transhydrogenase (NADTRHD), transketolase (TKT2) and NADP transhydrogenase (THD2).

In phase I, only NADTRHD is active, with zero fluxes for both TKT2 and THD2. The enzymatic reaction NADTRHD functions to convert NADPH into NADH. Thus in phase I, it is likely that the amount of NADPH produced exceeds the required amount for biosynthesis; NADTRHD is thus activated to consume the surplus NADPH.

In phase II, an on/off swap occurs between NADTRHD and TKT2 while THD2 still remains silent. We infer that in this phase, NADPH produced satisfies the demand, but the amount of carbon flowing into the PP pathway surpasses the rate of the carbon withdrawal (for the synthesis of biomass precursors). Therefore, TKT2 is activated to direct the extra amount of four-carbon and five-carbon compounds back to the glycolysis.

In phase III, THD2 is finally switched on and becomes significantly active in the high-growth-rates region. TKT2 increases progressively while NADTRHD remains silent. It is presumed that in this phase, as the growth rate becomes higher, more NADPH is required for biomass synthesis. NADP transhydrogenases (THD2) is activated to produce NADPH needed in rapid growth. The surplus carbon flux in the PP pathway, which might result from the high glucose uptake rate at a high growth rate, is directed back to glycolysis via TKT2.

It would be desirable to verify the theoretical prediction of the evolution of *PPP*% with experimental measurements, which unfortunately have not been widely reported in the literature. Nevertheless, Fig. 4b shows a comparison with one set of experimental observations available [42], which suggests a good degree of qualitative similarity.

### Adjusting cellular energy demand improves the prediction of biomass yield

**Fig. 5:**
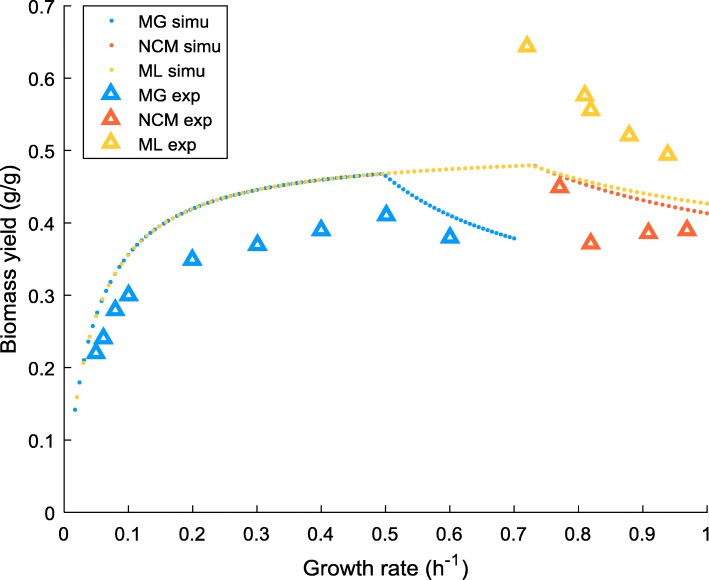
Comparison of the biomass yield between model predictions with nominal energy demand and the experimental data. “-nom” refers to nominal, the default energy demand specified in the core model. Biomass yield is calculated as gram biomass produced per gram substrate consumed. *uPPP*% was set to 35% for all strains. Experimental data were obtained from the same sources [3, 16, 41] of the acetate data shown in Fig. 1. simu – simulation results, exp. – experimental data

Although combining the PAT constraint with the core model succeeded in predicting the rates of acetate production, the accuracy in biomass yield varied and was especially unsatisfactory for ML strain (Fig. 5). A similar deficiency in yield prediction was also reported in [28]. Focusing on the yield, two features can be observed: (i) in the overflow region, for a fixed growth rate (associated with an acetate excretion rate) the biomass yield for ML is higher than MG and NCM; and (ii) the rate of the drop in yield (i.e. the slope) of ML is sharper than the other two strains.

Intuitively, feature (i) suggests that in ML, the amount of energy required per unit mass of biomass formation should be less than NCM or MG. Therefore we collected the growth data of ML and remodelled the cellular energy demand (see Methods).

It is worth noting that for ML, the negative value of M (Table 1) clearly indicates a constrained applicable range of the maintenance parameters, i.e. valid only within the overflow region. As growth rate decreases, if M stays unchanged, the overall energy consumption (Eq. (16)) will drop to a negative value, which is clearly not biologically feasible. This then implies a certain degree of nonlinearity in the global relationship between (total or maintenance) energy requirement and growth rate. Such proposition was previously referred to as “varied non-growth-associated maintenance” [3]. Non-linearity in energy consumption manifesting before and after the onset of the overflow metabolism has also been observed and discussed in a recent work [44].

### Model prediction of biomass yield with adjusted energy demand

**Fig. 6:**
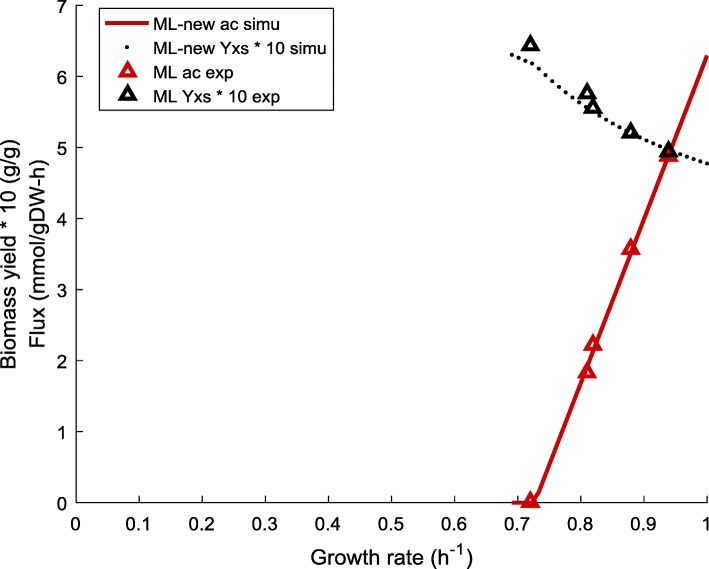
Model prediction of acetate production and biomass yield for ML with adjusted energy demand compared with experimental data. “-new” refers to the adjusted energy demand. Simulation was done with adjusted energy demand (Table 1, M,N for ML) and updated proteomic cost parameters. Biomass yields are shown as ten times of the original value to unify the order of magnitude between different types of data. *uPPP*% was set to 35%. Experimental data was obtained from Table 7 in [3]. ac – acetate flux, Yxs – biomass yield, simu – simulation results, exp. – experimental data

We first re-estimated the set of proteomic cost parameters for ML with the adjusted energy demand (see Table 1 and Methods). Applying updated values of ${w}_f^{\ast }$, ${w}_r^{\ast }$ and *b*^∗^ together with the adjusted maintenance energy, our model is now able to effectively capture the unique trend of biomass yield for ML, without any compromise in the accuracy of predicting acetate excretion (Fig. 6). Simulation results for ML with adjusted energy demand are referred to as “ML-new”.

It is worth noting that, our model also succeeds in matching the elevated reduction in the yield of ML in the overflow region as the growth rate increases. This captured trend appears to originate from the low energy demand of ML. Approximately, the yield reduction rate can be considered as being proportional to the ratio of the increase in the acetate excretion (*ac*_2_ − *ac*_1_) and the increase in glucose uptake rate (*glc*_2_ − *glc*_1_), while the growth rate rises from *λ*_1_ to *λ*_2_:

21 $$ yield\ reduction\ rate\propto \frac{ac_2-{ac}_1}{glc_2-{glc}_1}, for\ {\lambda}_1\to {\lambda}_2\ \left({\lambda}_2>{\lambda}_1\right) $$

NCM and ML exhibit similar acetate excretion rates, hence a similar value in “*ac*_2_ − *ac*_1_”. However, the energy demand per unit growth of ML is much lower than NCM, which means that with a similar increase in acetate production, the increase in substrate intake (i.e. *glc*_2_ − *glc*_1_) for ML will be lower than NCM to achieve a given increment in the growth rate. According to Eq. (21), the yield reduction rate of ML will thus be higher than NCM.

### Impact of the adjusted energy demand on ${w}_r^{\ast }-{w}_f^{\ast }$ and ${b}^{\ast }-{w}_f^{\ast }$ relationships

To investigate the impact of the change in cellular energy demand on the linear relationships of ${w}_f^{\ast }$, ${w}_r^{\ast }$ and *b*^∗^ of ML-new, we recalculated constants *k*_*f*_, *v*_*f*, 0_, *k*_*r*_ and *v*_*r*, 0_ at different *uPPP*% values (25, 35 and 40%) to update the linear equations describing ${w}_r^{\ast }-{w}_f^{\ast }$ line and ${b}^{\ast }-{w}_f^{\ast }$ (Eqs. (11) and (12)). The resulting ${w}_r^{\ast }-{w}_f^{\ast }$ lines for ML-new are plotted in Fig. 2, together with the results obtained earlier for MG/NCM/ML with nominal energy demand.

The switch to the adjusted energy demand makes the ${w}_r^{\ast }$ value for ML-new much higher than that of ML-nom, the latter being rather close to those of the MG-nom and NCM-nom. This implies that the adjustment of the energy demand of ML leads to an enlarged gap in the proteomic efficiency between respiration and fermentation.

**Fig. 7:**
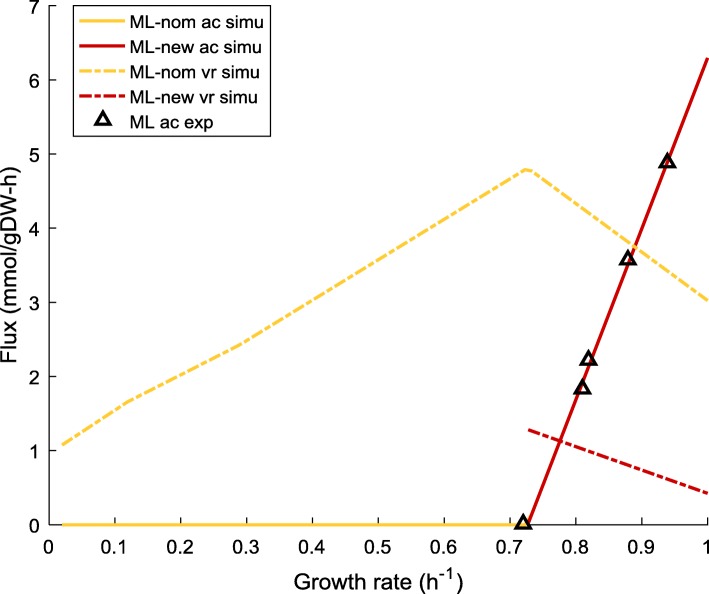
Comparison of the predicted respiration and acetate fluxes between ML-new and ML-nom. “-nom” refers to nominal, the default energy demand specified in the core model. “-new” refers to the adjusted energy demand. The predicted rates of acetate production for ML-nom and ML-new are completely overlapped with each other. *uPPP*% was set to 35% for both strains. Data source of acetate excretion is shown in Fig. 1. ac – acetate flux, vr – respiration flux, simu – simulation results, exp. – experimental data

The similarity among MG-/NCM−/ML-nom has already been discussed in the previous section. Here we mainly focus on the discrepancy with ML-new. We found that both the slope and intercept of ${w}_r^{\ast }-{w}_f^{\ast }$ line for ML-new are about three times larger than ML-nom (as shown in Additional file 1: Table S4). The dramatic changes in the slope and intercept of ML-new predominantly result from the reduction in the respiratory flux *v*_*r*_ when applying the adjusted energy demand (see Fig. 7 and Additional file 2: Figure S3).

The link between the drop in *v*_*r*_ and the increase in ${w}_r^{\ast }$ has been discussed in the previous section. The results presented herein indicate that it is the energy demand that plays a major role in distinguishing the ${w}_r^{\ast }-{w}_f^{\ast }$ relationship between different strains, not the *uPPP*% or the acetate excretion pattern.

**Fig. 8:**
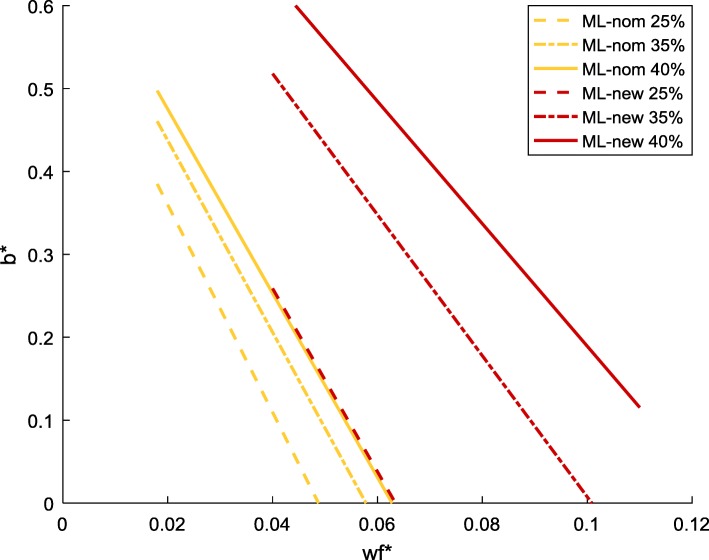
Comparison of the ${b}^{\ast }-{w}_f^{\ast }$ relationship between ML-nom and ML-new at different *uPPP*% levels. “-nom” refers to nominal, the default energy demand specified in the core model. “-new” refers to the adjusted energy demand. *uPPP%* was set to 25, 35 and 40% for both strains

Applying the adjusted energy demand also has an impact on the relationship between *b*^∗^ and ${w}_f^{\ast }$. As shown in Fig. 8, the ${b}^{\ast }-{w}_f^{\ast }$ lines are significantly right-shifted when the model is changed from ML-nom to ML-new (i.e. red lines are located in a much right area than yellow lines). Given the identical pattern of acetate excretion between ML-nom and ML-new (as both predicted the same set of experimental data), the amount of energy produced through fermentation remains unchanged. For ML-new, as the energy demand per unit of growth is much lower than that of the nominal strain, the respiratory flux *v*_*r*_ must decrease significantly to avoid energy overproduction, as confirmed in Fig. 7. Although the value of ${w}_r^{\ast }$ for ML-new is higher than that for ML-nom (for a given value of ${w}_f^{\ast }$, Fig. 2), the value of the product ${w}_r^{\ast }{v}_r$ for ML-new still becomes lower (as the increase in ${w}_r^{\ast }$ is not able to compensate for the sharp drop in *v*_*r*_). With no change in ${w}_f^{\ast }{v}_f$ between ML-new and ML-nom (for a given ${w}_f^{\ast }$), Eq. (6) again dictates *b*^∗^ to become higher for ML-new than ML-nom, hence the right-shifting of the ${b}^{\ast }-{w}_f^{\ast }$ lines.

In the case of ML-nom, it was shown earlier in Fig. 3 that the increase in *uPPP*% would lead to a reduction of *v*_*r*_, which in turn would lead to an increase in *b*^∗^ or right-shifting of the ${b}^{\ast }-{w}_f^{\ast }$ line. Now for ML-new, an enlarged gap between the ${b}^{\ast }-{w}_f^{\ast }$ lines at different *uPPP*% levels is observed compared to the case of ML-nom. This implies that the effect of *v*_*r*_ reduction due to the increase in *uPPP*% is more pronounced with the adjusted energy demand.

## Discussion

### Comparison with relevant models

In the study by Basan et al. [16], which has been the basis of the PAT constraint formulated in our model, the application of a constraint on proteome fractions (similar to Eq. (4)) with parameters derived from measured protein abundances was able to accurately predict the patterns of acetate excretion for *E. coli* under different growth conditions, when coupled with a simple energy balance equation. In this work, we have embedded the PAT into the core metabolic model of *E. coli*, taking the advantage of the latter in offering more rigorous modelling of intracellular mass and energy balances. Furthermore, the constraint-based metabolic model allows prediction of detailed metabolic fluxes as opposed to merely acetate production, which could provide more insights about metabolic pathways in connection with the overflow metabolism and pave the way for investigating acetate excretion in junction with possible manipulations of the metabolic network.

In addition, the respiratory flux in Basan’s work was associated with the carbon dioxide produced in respiration, termed *J*_*CO*2, *r*_, whose value was deduced by subtracting the fermentation-dependent CO_2_ and the growth-dependent CO_2_ from the total CO_2_ production. As such, *J*_*CO*2, *r*_ could not directly correspond to a specific flux in the metabolic network. In our model, the respiratory flux directly refers to a specific flux within the TCA cycle (AKGDH), which appears to be a convenient choice when the PAT is embedded into FBA. Using a constraint-based metabolic model that includes the TCA cycle with a reasonable level of detail, the respiration flux can be directly resolved via FBA, without the need for multi-step calculation along with different levels of assumptions and uncertainties.

Another important comparison we would like to make is with the recently developed model – CAFBA. It was mentioned in the Background section that unlike CAFBA which considers the proteomic cost of every individual reaction in metabolic network, the PAT constraint in our model follows the treatment of Basan et al.’s work and quantifies the proteomic costs at the pathway level. This simplification allows the model to explicitly incorporate the differential proteomic efficiencies of fermentation and respiration that are proven (in both Basan et al. and this work) to govern the flux split between the two pathways. This simplicity comes at the cost of the limited utility of our model: it is intended to be used only for predicting the interplay between the excretion of acetate (or other fermentation products) and the growth rate during overflow metabolism, not other effects of stressed resource allocation.

The proteome allocation constraint in CAFBA includes a C-sector (via a term expressed as *w*_*c*_*v*_*c*_) representing the proteome requirement for uptaking carbon source, which is not explicitly considered in this work. We have ignored this sector as the carbon overflow occurs only at the high growth rate region, where *w*_*c*_ (proteomic cost of the C*-*sector) approaches zero at high substrate uptake rates, as shown in CAFBA [28]. In this region, the low value of *w*_*c*_ makes the C-sector negligible compared with other proteome sectors. At low growth rate region, the value of *w*_*c*_ becomes significant, however no acetate is excreted in this region, where the equality relationship in the PAT constraint in our work becomes inactive so that the significance of the C-sector becomes irrelevant.

As for the prediction of biomass yield, CAFBA noticed the difficulty in predicting the biomass yield of ML308. In this work, we have found that it is the cellular energy demand that significantly affects the FBA prediction of biomass yield. After replacing the default energy demand with data reported specifically for ML308 strain, our model was able to produce an accurate prediction. Therefore, we consider that it is important to carry out necessary adjustment to the cellular energy demand when applying such a constraint-based modelling approach to specific strains.

### Parameterisation of the proteome allocation constraint

The modelling approach proposed in this work can be considered as “halfway” between the coarse-grained proteome allocation model of Basan et al. [16] and the FBA models that incorporate reaction-level resource allocation constraints such as CAFBA [28] and FBAwMC [21]. In CAFBA, the proteome constraint involves ~ 1000 proteomic cost parameters (*w*_*i*_) for a genome-scale model. Similarly in FBAwMC, a large number of crowing coefficients need to be specified. In both cases, the existence of numerous cost parameters originates from associating the resource cost with individual reactions. These parameters conceptually have a clear biological meaning and in principle can be determined experimentally by e.g. proteome measurements or extensive enzyme assays. However, in practice, it has appeared to be difficult to reliably obtain precise values for all the parameters, especially for different strains growing at different growth rates or conditions. In fact, instead of pursuing the exact values for all the individual parameters, CAFBA focused on applying the average value of the proteome fraction invested per unit flux, termed as 〈*w*〉, to capture the key flux pattern, along with evaluating the impact of possible heterogeneous values of the proteome parameter *w*_*i*_ on the model prediction. Similarly, FBAwMC [21, 22] also appears to encounter a certain degree of “randomness” of its crowding coefficients due to the unknown enzyme kinetics and/or turnover numbers. Subsequently, molecular-crowding-based modelling normally treats this “randomness” as noise, where the crowding coefficients are chosen randomly from a distribution of crowding coefficients [45] or the majority of the crowding coefficients are estimated from a limited number of known enzyme turn-over (k_cat_) values [11].

In this work, we have intended to formulate a constraint with a greatly reduced number of proteomic cost parameters, while still capturing the essence of constrained cellular resource allocation. This is achieved by formulating the proteome allocation constraint at the pathway (as opposed to reaction) level. The proposal is the concise Eq. (6) (${w}_f^{\ast }{v}_f+{w}_r^{\ast }{v}_f+{b}^{\ast}\lambda \le 1$), involving only three proteomic cost parameters (representing proteomic efficiencies of fermentation, respiration and biomass synthesis pathways). In principle, these parameters can be obtained through the direct measurement of protein abundances, following an approach similar to that adopted by Basan et al.’s work [16]. However, in the current study, we attempted to parameterise this constraint using widely available growth data from cell culturing experiments, in particular growth rate and acetate production rate. It should be noted that cell culturing experiments often yield relatively simple data sets with measurements of a few process variables. It is infeasible to use such data sets to determine a large number of proteomic cost parameters encountered in a proteome constraint expressed at the individual reaction level. Even with the pathway-level constraint adopted in this work, our results show that cell growth and acetate production measurements (alone) cannot uniquely determine the three parameters, but two linear relationships between these parameters can be derived (Eqs. (11) and (12)).

Furthermore, our model shows that it is the two linear relationships (but not the absolute values) of the proteomic cost parameters that allow an accurate prediction of the overflow metabolism. We thus speculate that for a FBA-based model, the ability of capturing the overflow behaviour is rendered by (i) an extra constraint representing the constrained proteomic resources and (ii) certain relations or relative magnitudes of the proteomic cost parameters embedded in the proteome constraint. In reality, the proteomic efficiencies of the metabolic pathways may vary (within a certain range), at different points in time or between cells in a population which often exhibits heterogeneity [28]. However, as long as the specific relations or relative magnitudes of these efficiencies are maintained, one can expect that the overflow behaviour will emerge.

### Applicability of the linear formulation of the proteomic cost

As indicated in the earlier section, our formulation of the proteomic cost (Eqs. (2a) and (2b)) reflects the observed linear dependency between proteome fraction and growth rate [30–32]. Combining this linear dependency with the assumption that the flux processed by a proteome sector *i* is proportional to the growth rate-dependent component of the associated proteome fraction [30], we have related *ϕ*_*i*_ linearly with the flux it carries. A similar model is also adopted in CAFBA [28], where the linear proteome-flux relation is derived on the assumption that the substrate concentration is proportional to the flux.

Note that our model is intended specifically for predicting the steady-state overflow metabolism in *E. coli* under glucose-limited conditions. In some other circumstances, observations not conforming to this relatively simple model have been reported. For example, Goel and his co-workers found hardly any changes in protein levels in anaerobic slow-growing *Lactococcus lactis* chemostats*,* when the cell shifted from a high yield metabolic mode to a low yield metabolic mode with an increased growth rate [46]. In this case, although the metabolic shift in *L. lactis* is similar to the overflow metabolism observed in *E. coli*, proteome allocation did not seem to accompany the changes in metabolic fluxes. In a study on yeast’s transient transcript, enzyme and metabolite responses under metabolic perturbation, it is revealed that the reaction rates are jointly regulated by enzyme capacity and metabolite concentration due to the cell’s tendency in sacrificing the local metabolite homeostasis to maintain fluxes and global metabolite homeostasis upon enzyme perturbation [47]. Another yeast-based study also suggests that changes in individual flux are predominantly regulated by the levels of metabolites, not enzymes [48].

The above-mentioned experimental observations suggest that the linear proteome-flux relationship modelled in this work might not be applicable to those circumstances. We hypothesise that this might be at least partially due to the differences between *E. coli* (the target organism of our model) and the organisms with which those observations were made. Besides, our model has been developed to describe the relationship between (i) the observed steady-state global proteome configuration and (ii) the growth rate or the corresponding flux, drawing on evidences and hypothesises from several previous studies [16, 28, 30, 32]. Such a model, being global and coarse-grained, is not intended to represent delicate regulatory mechanisms responsible for the transient metabolic changes to maintain cellular homeostasis under perturbations, and might not be suitable for revealing the local regulatory insights on the key factors dictating the individual reaction rates.

## Conclusions

With three different *E. coli* strains, we have evaluated a new model that integrates a previously proposed proteome allocation theory (PAT) into the constraint-based modelling approach – flux balance analysis (FBA), which predicts the distribution of carbon fluxes between fermentation and respiration due to the differential proteomic efficiencies of the two energy biogenesis pathways. Using a simple proteome allocation constraint, our model allows the accurate prediction of acetate production at different steady state growth rates during overflow conditions (with sufficient oxygen and glucose). The model involves three pathway-level proteomic cost parameters linearly interrelated by two equations, which is the consequence of (i) the assumed linear dependency of proteomic costs and the growth rate and (ii) the experimentally observed linear correlation between the fermentation or respiration flux and the growth rate. The non-unique optimal values of the three parameters, or the two linear relationships between them, could be obtained by fitting the model to experimentally measured acetate excretion rates at specific growth rates.

The linear relationships between the parameters were shown to be affected, in varying degrees, by (i) the acetate excretion pattern, (ii) the assumed upper limit of the substrate carbon diverting into PP pathway and (iii) the cellular energy demand. The proteomic cost of the fermentation pathway was estimated always to a lower value than that of the respiration pathway, i.e. ${w}_f^{\ast }<{w}_r^{\ast }$. The proteomic cost of the biomass synthesis sector was estimated to be higher in a slow-growing strain that excretes acetate at a lower growth rate, in comparison with the other two fast-growing strains, i.e. ${b}_{\mathrm{MG}}^{\ast }>{b}_{\mathrm{NCM}/\mathrm{ML}}^{\ast }$. The estimated values of ${w}_f^{\ast }$ and *b*^∗^both meet qualitatively the expectation from a biological point of view. Furthermore, the relationship between the proteomic efficiencies of fermentation and respiration, i.e. the ${w}_f^{\ast }-{w}_r^{\ast }$ line, was shown to change between different strains most significantly with the cellular energy demand rather than with the pattern of acetate excretion. This ${w}_f^{\ast }-{w}_r^{\ast }$ relationship remained relatively stable when the upper bound of the portion of substrate carbon flowing into PP pathway (*uPPP*%) varied in the modelling studies. On the other hand, the increase of *uPPP*% was shown to lead to a visible increase in the estimated proteomic cost of the biomass synthesis sector *b*^∗^, which mathematically results from a reduction in the predicted respiration flux.

Finally, and as a general point for constraint-based models, cellular energy demand appeared to have a major impact on the predicted biomass yield; tuning the default energy demand with strain-specific data was shown to be critical in making simultaneously accurate predictions of biomass yield and overflow metabolism.

Overall, this work demonstrates the potential of combining a detailed metabolic model with a coarse-grained, pathway-level resource allocation constraint in producing quantitatively accurate predictions of the overflow phenomenon in *E coli*; similar modelling approaches that feature this type of combination may prove suitable also for other applications.

## Additional files

Additional file 1: Supplementary Tables S1-S6. (XLSX 26 kb)

Additional file 2: Supplementary text and Supplementary Figures S1-S17. (DOCX 229 kb)

## Abbreviations

FBA: Flux balance analysis
GAM: Growth-associated maintenance
NADTRHD: NAD transhydrogenase
NGAM: Non-growth-associated maintenance
PAT: Proteome allocation theory
PPP%: Pentose phosphate pathway ratio
THD2: NAD(P) transhydrogenase
TKT2: Transketolase
uPPP%: Upper bound of the pentose phosphate pathway ratio
