# Supplementary material for: Modelling overflow metabolism in Escherichia coli with flux balance analysis incorporating differential proteomic efficiencies of energy pathways
Source: BMC Syst Biol. 2019 Jan 10;13:3. doi: 10.1186/s12918-018-0677-4 (PMC6329140; doi:10.1186/s12918-018-0677-4)
Supplement: Supplementary file 2 — Supplementary text and Supplementary Figures S1-S17. (DOCX 229 kb) [file 12918_2018_677_MOESM2_ESM.docx]

Additional file 2

# 1. The core *Escherichia coli* metabolic model

We use the previously published core *Escherichia coli* metabolic model [1] to implement the PAT-based FBA model. The core model consists of 95 cellular reactions and 72 unique metabolites. The COBRA Toolbox [2] was used to load metabolic reconstruction with the addition of global regulations. In particular, the lower bound of the oxygen exchange flux (EX_o2(e)) and the glucose exchange flux (EX_glc(e)) were set to -1000 to mimic the aerobic-glucose conditions where aerobic formation of acetate (overflow metabolism) occurs. Reactions involved in glyoxylate shunt, gluconeogenesis and aerobic specific processes were determined to be off according to previous published regulatory rules for *E. coli* growing under aerobic-glucose conditions [3]. We also closed the exchange and synthesis fluxes of metabolites that were not reported by pertinent experiments [4–6]. The complete list of reactions that are determined to be off is given in Table S6 (Additional file 1). Reactions in glycolysis, TCA cycle, pentose phosphate pathway, acetate synthesis pathway and oxidative phosphorylation system were set to carry flux in the forward direction based on flux measurement [7]. The cellular objective is set to minimize substrate uptake rate (at fixed growth rate).

# 2. Integrating PAT to Flux Balance Analysis

To fuse the PAT constraint into FBA, we add an additional row to the $S$ matrix of the metabolic model with the proteomic efficiency parameters $w_{f}^{*}$,$w_{r}^{*}$ and $b^{*}$ at the column position corresponding to fermentation flux (ACKr), respiration flux (AKGDH) and the biomass flux (Biomass_Ecoli_core_w/GAM), respectively. Reaction names are consistent with the notation used in the *E. coli* core model. The right hand side of the constraint is fulfilled by introducing an auxiliary reaction DM_A

$w_{f}^{'}v_{f}+w_{r}^{'}v_{r}+b^{'}\lambda=DM\_A$ (S1)

The upper bound of DM_A is set to one

$w_{f}^{'}v_{f}+w_{r}^{'}v_{r}+b^{'}\lambda=DM\_A\leq1$ (S2)

# 3. Impact of PP pathway ratio on model parameters and predictions

As mentioned in the Methods, the portion of substrate carbon diverting into PP pathway can vary with different growth rates (achieve a maximum ~20%) and culture conditions [4,8]. Such uncertainty of the PP pathway flux motivates us to investigate the impact of adjusting the ratio of carbon flowing into the PP pathway to the total carbon intake on the model performance. In the main text (determination of Eq.(11) and Eq.(12)), we confirmed that, when fixing growth rate and acetate excretion flux, with minimizing glucose uptake as the objective, FBA solution was unique, which provides the foundation of using FBA solution of PP pathway fluxes for further analysis. Applying the experimentally determined acetate excretion - growth rate profile to FBA, a maximum level of PP pathway ratio (*PPP%*) was found for all tested species (~60%, as shown in Fig. S6-S8). The mean value of the maximum *PPP%* of the flux measurement and the *in silico* result is ~40%. Thus we specified different level of upper bound of PP pathway ratio (*uPPP%*) as 25%, 35% and 40%, along with the proteome allocation constraint to explore the impact of PP pathway uncertainty. Results show that, given a certain species, different uPPP% leads to different values of the $k_{r}$ and $v_{r,0}$ (the slope and intercept of the respiration line) i.e. respiration flux changes with *uPPP%*, thus rendering different Eq.(11) and Eq,(12). For each *uPPP%*, we obtain a collection of mathematically equivalent $w_{f}^{*}$, $w_{r}^{*}$ and $b^{*}$. Fig. 2 and Fig. 3 shows that changes in PP pathway ratio affect both $w_{r}^{*}$ and $b^{*}$ ($w_{f}^{*}$ was fixed as the independent variable), with the latter being affected slightly more significantly.

Furthermore, we confirmed that adopting any set of $w_{f}^{*}$, $w_{r}^{*}$ and $b^{*}$ showed in Fig. 2 and Fig. 3 as the PAT constraint provides non-differentiable model prediction on acetate production. Therefore, the variation of PP pathway ratio, as long as is within a reasonable range (e.g. ~ 20% reported in [4,8]), will not violate the model prediction of the overflow metabolism. It is worth noting that, biologically, PP pathway possesses much less proteome fraction compared with the lower part of glycolysis (~ 1/4) [5], making whatever modulations in the upper glycolysis (due to alternative pathway) insignificant to the overall proteome allocation, coinciding our finding of the relation between *uPPP%* and the overflow metabolism.

# 4. Supplementary Figure S1-S8

Fig. S1 Comparison of the $b^{*}-w_{f}^{*}$ relationship between MG, NCM and ML with nominal energy demand. $uPPP\%$ was set at 25% 35% and 40% for each strain.

Fig. S2 Model predictions of acetate production (ac) and biomass yield (Yxs) for MG and NCM with adjusted maintenance energy parameters. Maintenance parameter M was adjusted to 16 and 25 for MG and NCM, respectively. $uPPP\%$ was set to 35% for both MG and NCM strains; other *uPPP%* values generate similar results.

Fig. S3 Comparison of the simulation values of the respiration flux $\boldsymbol{v}_{\boldsymbol{r}}$ between ML-nom and ML-new. *uPPP%* was set to 25, 35 and 40% for each strain.

b)

a)

Fig. S4 a) Simulation results of the PP pathway fluxes at different growth rates for MG strain. b) Comparison of the model prediction and the experimental data of the portion of substrate carbon diverting into PP pathway. uPPP% was set to 25%. Nominal energy demand.

b)

a)

Fig. S5 a) Simulation results of the PP pathway fluxes at different growth rates for MG strain. b) Comparison of the model prediction and the experimental data of the portion of substrate carbon diverting into PP pathway. *uPPP%* was set to 40%. Nominal energy demand.

MG

Fig. S6 Change of PP pathway ratio against growth rate for MG strain when *uPPP%* set to 100%. Nominal energy demand.

NCM

Fig. S7 Change of PP pathway ratio against growth rate for NCM strain when *uPPP%* set to 100%. Nominal energy demand.

ML

Fig. S8 Change of PP pathway ratio against growth rate for ML strain when *uPPP%* set to 100%. Nominal energy demand.

# 5. Supplementary Figure S9-S17

Figures contained in this section show that arbitrarily chosen values of $w_{f}^{*}$ yield identical simulation results (rates of acetate production and the tca flux). tca flux is termed as “vr” in the main text. Both of them are obtained from the simulated flux of the enzymatic reaction AKGDH, a key reaction in the TCA cycle to represent the extent of the reparation.

Fig. S9 Model prediction of acetate and tca flux at $w_{f}^{*}=0.03$ for MG strain.

Fig. S10 Model prediction of acetate and tca flux at $w_{f}^{*}=0.05$ for MG strain.

Fig. S11 Model prediction of acetate and tca flux at $w_{f}^{*}=0.07$ for MG strain.

Fig. S12 Model prediction of acetate and tca flux at $w_{f}^{*}=0.01$ for ML strain.

Fig. S13 Model prediction of acetate and tca flux at $w_{f}^{*}=0.03$ for ML strain.

Fig. S14 Model prediction of acetate and tca flux at $\boldsymbol{w}_{\boldsymbol{f}}^{*}=\boldsymbol{0}.\boldsymbol{04}$ for ML strain.

Fig. S15 Model prediction of acetate and tca flux at $w_{f}^{*}=0.01$ for NCM strain.

Fig. S16 Model prediction of acetate and tca flux at $w_{f}^{*}=0.03$ for NCM strain.

Fig. S17 Model prediction of acetate and tca flux at $w_{f}^{*}=0.05$ for NCM strain.

# References

1. Orth JD, Palsson BØ, Fleming RMT. Reconstruction and Use of Microbial Metabolic Networks: the Core Escherichia coli Metabolic Model as an Educational Guide. EcoSal Plus [Internet]. 2010;4. Available from: http://www.asmscience.org/content/journal/ecosalplus/10.1128/ecosalplus.10.2.1

2. Becker SA, Feist AM, Mo ML, Hannum G, Palsson BØ, Herrgard MJ. Quantitative prediction of cellular metabolism with constraint-based models: the COBRA Toolbox. Nat Protoc [Internet]. 2007;2. Available from: http://dx.doi.org/10.1038/nprot.2007.99

3. Feist AM, Henry CS, Reed JL, Krummenacker M, Joyce AR, Karp PD, et al. A genome-scale metabolic reconstruction for Escherichia coli K-12 MG1655 that accounts for 1260 ORFs and thermodynamic information. Mol Syst Biol [Internet]. 2007;3. Available from: http://msb.embopress.org/cgi/doi/10.1038/msb4100155

4. Nanchen A, Schicker A, Sauer U. Nonlinear dependency of intracellular fluxes on growth rate in miniaturized continuous cultures of Escherichia coli. Appl Environ Microbiol. Am Soc Microbiol; 2006;72:1164–72.

5. Basan M, Hui S, Okano H, Zhang Z, Shen Y, Williamson JR, et al. Overflow metabolism in Escherichia coli results from efficient proteome allocation. Nature [Internet]. 2015;528:99–104. Available from: http://www.nature.com/doifinder/10.1038/nature15765

6. Holms H. Flux analysis and control of the central metabolic pathways in Escherichia coli. FEMS Microbiol Rev. 1996;19:85–116.

7. Fischer E, Sauer U. A novel metabolic cycle catalyzes glucose oxidation and anaplerosis in hungry Escherichia coli. J Biol Chem. ASBMB; 2003;278:46446–51.

8. Sauer U, Canonaco F, Heri S, Perrenoud A, Fischer E. The soluble and membrane-bound transhydrogenases UdhA and PntAB have divergent functions in NADPH metabolism of Escherichia coli. J Biol Chem. ASBMB; 2004;279:6613–9.
